# Changes in gut bacterial populations and their translocation into liver and ascites in alcoholic liver cirrhotics

**DOI:** 10.1186/1471-230X-14-40

**Published:** 2014-02-24

**Authors:** Sari Tuomisto, Tanja Pessi, Pekka Collin, Risto Vuento, Janne Aittoniemi, Pekka J Karhunen

**Affiliations:** 1 Department of Forensic Medicine, University of Tampere, School of Medicine, Medisiinarinkatu 3, 33014 Tampere, Finland; 2 Department of Gastroenterology and Alimentary Tract Surgery, Tampere University Hospital, Tampere, Finland; 3 Fimlab Laboratories, Pirkanmaa Hospital District, Biokatu 4, 33520 Tampere, Finland

**Keywords:** Alcoholic liver cirrhosis, Gut microbiota, RT-qPCR, Bacterial translocation, Microbiology, CD14

## Abstract

**Background:**

The liver is the first line of defence against continuously occurring influx of microbial-derived products and bacteria from the gut. Intestinal bacteria have been implicated in the pathogenesis of alcoholic liver cirrhosis. Escape of intestinal bacteria into the ascites is involved in the pathogenesis of spontaneous bacterial peritonitis, which is a common complication of liver cirrhosis. The association between faecal bacterial populations and alcoholic liver cirrhosis has not been resolved.

**Methods:**

Relative ratios of major commensal bacterial communities (*Bacteroides* spp., *Bifidobacterium* spp., *Clostridium leptum* group*, Enterobactericaea* and *Lactobacillus* spp.) were determined in faecal samples from post mortem examinations performed on 42 males, including cirrhotic alcoholics (n = 13), non-cirrhotic alcoholics (n = 15), non-alcoholic controls (n = 14) and in 7 healthy male volunteers using real-time quantitative PCR (RT-qPCR). Translocation of bacteria into liver in the autopsy cases and into the ascites of 12 volunteers with liver cirrhosis was also studied with RT-qPCR. CD14 immunostaining was performed for the autopsy liver samples.

**Results:**

Relative ratios of faecal bacteria in autopsy controls were comparable to those of healthy volunteers. Cirrhotics had in median 27 times more bacterial DNA of *Enterobactericaea* in faeces compared to the healthy volunteers (p = 0.011). *Enterobactericaea* were also the most common bacteria translocated into cirrhotic liver, although there were no statistically significant differences between the study groups. Of the ascites samples from the volunteers with liver cirrhosis, 50% contained bacterial DNA from *Enterobactericaea*, *Clostridium leptum* group or *Lactobacillus* spp.. The total bacterial DNA in autopsy liver was associated with the percentage of CD14 expression (p = 0.045). CD14 expression percentage in cirrhotics was significantly higher than in the autopsy controls (p = 0.004).

**Conclusions:**

Our results suggest that translocation of intestinal bacteria into liver may be involved as a one factor in the pathogenesis of alcoholic liver cirrhosis.

## Background

Liver cirrhosis is the irreversible end stage of chronic liver disease. In Western countries more than 90% of cirrhosis is caused by excessive ethanol consumption. However, only a minority of alcohol abusers ever develop liver cirrhosis during their lives. Our group has previously reported a link between alcoholic liver cirrhosis and bacterial recognition receptor *CD14* genetics [1] supporting the hypothesis of the involvement of intestinal bacteria and their endotoxins in the pathogenesis of cirrhosis [2]. This observation has been confirmed by other groups [3,4].

In healthy individuals, normal gut microbiota consists mainly of bacteria belonging to the *Clostridium coccoides* (cluster XIVa) and *Clostridium leptum* group (cluster IV), *Bacteroides* spp., *Bifidobacterium* spp. and *Enterobacter* spp. [5,6]. *Enterobacter* spp. belongs to a large *Enterobactericaea* family consisting of several gram-negative bacteria [7]. The liver is continually exposed to gut derived bacteria and bacterial components because ca. 70% of its blood supply is from the portal vein, which is the direct venous outflow of the intestine [8]. Chronic alcohol abuse has been shown to change intestinal bacterial population by decreasing the numbers of *Clostridium* (gram-positive, anaerobe) and *Bacteroidetes* (gram-negative, anaerobe) and increasing the numbers of aerobic *Proteobacteria* (gram-negative, facultatively or obligately anaerobic) [9].

Alcohol can cause leakage of intestinal cell junctions [10]. Gram-negative bacteria [11] and their endotoxins [12] may then translocate through portal blood into the liver and induce inflammation leading to fibrosis and cirrhosis in genetically susceptible individuals [1]. The quantities of *Bacteroides* spp. and *Bifidobacterium* spp. have been reported to be decreased and those of *Enterobacter* spp. and *Clostridium* spp. to be increased in the faeces of cirrhotics [13,14], but it is not known whether these bacteria may translocate into the liver.

Spontaneous bacterial peritonitis is a complication of cirrhosis. It is thought to be due to bacterial overgrowth in the gut and their translocation into the ascites [15]. Its frequency has been 7% in cirrhotic patients with ascites [15]. Bacterial culture is unreliable, and the diagnosis is based on a polymorphonuclear cell count of 250/mm^3^ or more in the ascites fluid, regardless of the result of received by bacterial culturing. Bacterial contamination may be far more common than manifest in spontaneous bacterial peritonitis or positive bacterial culture. It is possible that bacteria which may be associated with the pathogenesis of cirrhosis may also be present in ascites when measured by molecular biological methods instead of conventional culturing.

We have previously reported that liver samples from autopsies can be reliably used in bacteriological analyses up to 5 days post mortem [16], and that the faecal bacterial composition of certain bacteria remains stable during that period [17]. In an earlier paper we also have shown in the present autopsy series that bacteria can be detected by culturing and qPCR in mesenteric lymph nodes but also in the portal vein and liver [16]. The aim of this study was to characterize changes in intestinal microbiota (*Bacteroides* spp., *Bifidobacterium* spp., *Clostridium leptum* group*, Enterobacteriacaea,* and *Lactobacillus* spp.) in alcoholic liver cirrhotics compared to alcoholics without cirrhosis, non-alcoholic autopsy controls and in healthy volunteers, and to study the translocation of bacteria into liver and ascites samples. In order to confirm the significance of our bacterial findings, the activity of bacteria recognizing receptor CD14 was studied by immunostaining of autopsy liver samples and correlated with the presence and amount of bacterial DNA in liver.

## Methods

The present study comprised a prospective autopsy series of 42 males at the Department of Forensic Medicine of the University of Tampere, and 7 male healthy volunteers and 12 male clinical volunteers with alcoholic liver cirrhosis with ascites at the Department of Gastroenterology and Alimentary Tract Surgery (Table 1). From the routine autopsies, we selected cases with alcoholic cirrhosis (n = 13), alcoholics (n = 15) without liver cirrhosis and a control series (n = 14) of non-alcoholic men. Criteria for including autopsies were: out-of-hospital death, male sex, age over 18 years, time elapsed post mortem 5 days or less, time interval between death and storage of the body in the mortuary less than 24 hours, intact middle torso and bowel, no signs of bacterial infections or visible wounds or necrosis and no signs or reports of drug addiction. None of the study subjects were reported to have taken antibiotics for 2 weeks prior to sampling. Two of the 42 men died of alcohol intoxication and in 17 cases alcohol was a contributory factor for death. One subject died of alcoholic liver cirrhosis. The hospital records of the autopsy cases were available, and were scrutinized for mentions of alcohol use. The criterion for the definition of alcoholism or heavy alcohol consumption was a comment in the hospital records/police reports or alcoholism-related microscopic findings such as presence of alcoholic liver disease or cerebellar atrophy, along with positive post-mortem alcohol test. Of the 15 alcoholics, 11 had mentions of alcoholism in their documentation, 12 had positive post mortem alcohol test and 6 also had microscopic findings (fatty liver, cerebellar atrophy or chronic pancreatitis) of alcoholism.

**Table 1:** Demographic characteristics of the study subjects

	**Autopsy cases**			**Volunteers**		
	**Cirrhotics**	**Alcoholics**	**Non-alcoholic controls**	**Healthy controls**	**Liver cirrhosis**	**P-value**
N	13	15	14	7	12	
PM time mean	3.6	3.3	3.8			0.537
Age mean (range)	56 (39–77)	54 (34–77)	58 (18–86)	45 (26–57)	58 (39–73)	0.383
BMI mean (range	29.1 (19.6-41.9)	30.1 (20.4-42.1)	27.4 (18.4-43.6)	27.1 (20.8-37.2)	28.1 (19.7-39.2)	0.573
Cause of death						
Heart disease%	5 (38.5%)	3 (20.0%)	10 (71.4%)			0.007
Other disease%	5 (38.5%)	8 (53.3%)	1 (7.1%)			0.017
Non-natural death^1^%	3 (23.1%)	4 (26.7%)	3 (21.4%)			0.885

^1^suicide, accident, poisoning.

Written consent was obtained from the volunteers. None of the healthy volunteers drank more than 3 drinks (12 g of alcohol) per week. Volunteers with liver cirrhosis reported excessive alcohol consumption. None of the study subjects were reported to have taken antibiotics for 2 weeks prior to sampling.

Samples from rectum and liver were taken aseptically from the autopsy cases. The healthy volunteers provided samples after defecation. All faecal samples were frozen (−20°C) immediately after sampling and were transferred to −80°C until further processing. Ascites fluid samples were collected from patients during their hospital stay with sterile instruments.

Ascites fluid samples were drawn aseptically at the bedside from the volunteers with a diagnosis of alcohol-related cirrhosis, which was based on drinking history and clinical findings. Bacterial conventional culturing as well as albumin and polymorphonuclear measurements from ascites samples were performed.

Faecal samples were weighed to be 150 mg (wet weight). Bacterial DNA was extracted from the faecal samples using a commercial DNA extraction kit (Zymo Fecal DNA Kit (Zymo Research Corporation, Irvine, California, USA)) according to the instructions provided. DNA from liver, blood and ascites fluid samples was extracted using Zymo Bacterial/Fungal DNA Kit (Zymo Bacterial/Fungal DNA Kit (Zymo Research Corporation, Irvine, California, USA)).

The quantity of bacteria was determined by RT-qPCR using published oligonucleotide primers and probes for *Bacteroides* spp. [18], *Bifidobacterium* spp. [19], *Clostridium leptum* group (cluster IV) [19], and *Enterobacter* spp. [20]. The primers and probe for *Enterobacteriacaea* (Forward: GCGGTAGCACAGAGAGCTT, Reverse: GGCAGTTTCCCAGACATTACTCA, PROBE:6FAM-CCGCCGCTCGTCACC-BHQ), and *Lactobacillus* spp. (Forward: GCTAGGTGTTGGAGGGTTTCC, Reverse: CCAGGCGGAATGCTTAATGC, PROBE:6FAM- TCAGTGCCGCAGCTAA-BHQ) were designed and confirmed using BLAST with the National Centre for Biotechnology Information server (http://www.ncbi.nlm.nih.gov) and Ribosomal Database Project (http://rdp.cme.msu.edu/probematch/search.jsp). Specificity and cross reactivity of all the primers and probes were tested using bacteria from clinical samples and reference bacteria [16,21]. Total amount of bacteria was measured using universal bacterial primers and a probe [22]. Assays from faecal samples and from ascites were performed with AbiPrism 7000 HT Sequence Detection System (Taqman™, AppliedBiosystems, California, USA) in reaction volume of 20 μl in 96-well reaction plate under standard conditions with Taqman. Faecal samples were diluted 1:100. In the liver samples, assays were performed with AbiPrism 7900 HT Sequence Detection System (Taqman®, AppliedBiosystems) in a reaction volume of 5 μl. One micro litre of DNA was added into the reactions for the detection system. MasterMix was prepared using Taqman™ Environmental MasterMix adding at final concentrations of 1000 nM of each primer, and 250 nM of each fluorescence labelled probe. All amplifications and detections were carried out in duplicate or quadruplicate (in uncertain cases).

The relative amount of bacterial DNA in a sample was determined with comparative Ct method (ΔΔCt, ΔCt _sample_ – ΔCt _reference sample_) [23]. In autopsy faecal samples, mean ΔC values of the faecal samples of healthy volunteers were calculated and used as a reference. In ascites fluid and liver samples the blood sample from a healthy individual was used as a reference as previously described [16]. Bacterial positivity of liver and ascites samples was determined as previously described [16].

Histological samples from the right lobe of the liver were taken at autopsy, fixed in 10% formalin overnight and processed for CD14 (Leica Biosystems, Newcastle, United Kingdom) immunohistochemistry, using dilution of 1:250 with Autostainer LV-1 (Lab Vision Corporation, California, USA). The percentage of brown peroxidase positivity in each sample was calculated with the ImmunoRatio program [24] and average discolouration value was compared between different groups.

The study was approved by the Ethics Committee of Pirkanmaa Hospital District and the National Supervisory Authority for Welfare and Health (VALVIRA).

### Statistics

Due to the skewed variation within the bacterial measurements, logarithmic values were used for the calculations [IBM SPSS Statistics version 21 (IBM, New York, United States)]. ANOVA was used to measure significant differences between groups (healthy volunteers, alcoholic cirrhotics, alcoholics and non-alcoholic controls). When ANOVA showed a significant difference, pairwise comparisons with Post Hoc test with Least Significant Difference (LSD) corrections were made. With liver samples, Pearson’s Chi-square test was used.

## Results

**Figure 1:**
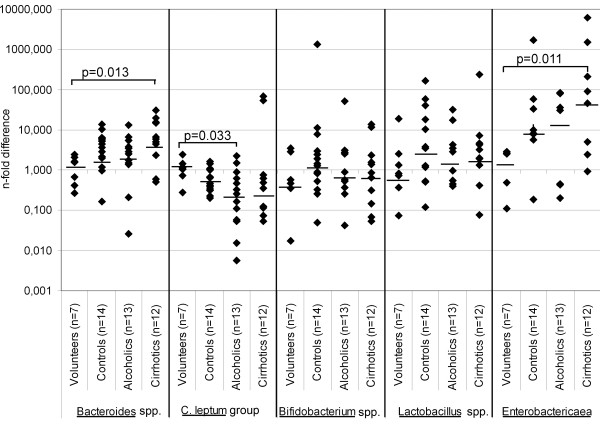
**Relative amounts (n-fold difference) of bacteria measured (*Bacteroides *spp., *C. leptum *group, *Bifidobacterium *spp., *Lactobacillus *spp., *Enterobactericaea*) in faecal samples of cirrhotic, alcoholic and autopsy control cases and in healthy volunteers.** Individual values are presented as diamond, median values with horizontal lines.

The median values of relative amounts of measured bacterial groups in the faeces of the autopsy controls were comparable to those in the healthy volunteers (Figure 1). Although the numbers in the groups were small, there were differences across study groups (healthy volunteers, controls, alcoholics and cirrhotics) in the relative amounts of *Bacteroides* spp. (p = 0.070) and *Enterobactericaea* (p = 0.056). Post Hoc analysis showed that males with cirrhosis had significantly more gram-negative *Bacteroides* spp. (p = 0.013) and gram-negative *Enterobactericaea* (p = 0.011) in autopsy faecal samples than did the healthy volunteers. Of *Enterobactericaea*, autopsy faecal samples from males with cirrhosis contained considerably more gram-negative *Enterobacter* spp. (p = 0.034) than those from alcoholics without liver cirrhosis (data not shown). When comparison was done against all other groups combined (alcoholics, control autopsies and healthy volunteers), the faeces of alcoholic cirrhotics contained statistically significantly more gram-negative *Enterobactericaea* (p = 0.037), *Enterobacter* spp. (p = 0.047) and *Bacteroides* spp. (p = 0.049). No differences were seen in the proportion of *Bifidobacterium* spp. and *Lactobacillus* spp. between different groups. Inter-individual variation was great in all bacterial measurements.

**Figure 2:**
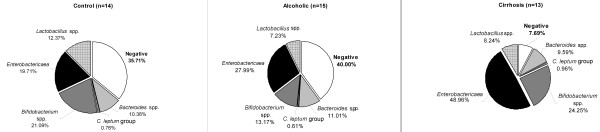
Percentages of bacteria found in the liver samples of autopsy cases (n = 42) with RT-qPCR.

Bacterial DNA positivity was in liver samples of cirrhotics 92%, alcoholics 60% and in the controls 64% (Figure 2). These differences did not reach statistical significance (p = 0.130, Pearson Chi Square). The most common bacterial DNA detected in the liver samples of cirrhotics was *Enterobactericaea* (49%) and *Bifidobacterium* spp. (24%). Differences between detected bacteria in the groups did not reach statistical significance; wide inter-individual variation was detected.

**Figure 3:**
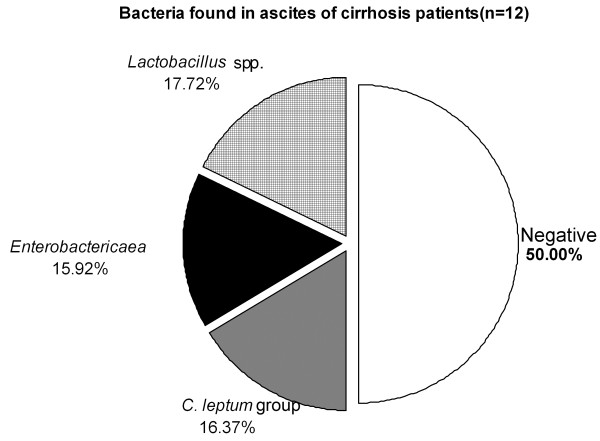
**Bacteria found in the ascites samples of volunteers with liver cirrhosis (n = 12) with RT-qPCR specific primers and probes.** A blood sample from a healthy volunteer was used as a reference.

Of the ascites fluid samples, 50% contained bacterial DNA. Of the bacteria measured *Lactobacillus* spp., *C. leptum* group and *Enterobactericaea* were found, whereas *Bifidobacterium* spp. and *Bacteroides* spp. were not amplified (Figure 3). In all 12 samples, the ascites leukocyte count was below 250 10E6/l, thus none of the patients fulfilled the criteria of spontaneous bacterial peritonitis. Ascites bacterial culturing was negative in each case.

**Figure 4:**
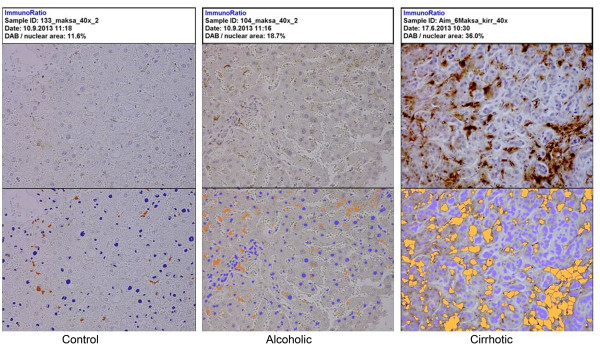
Immunohistological staining of CD14 as DAB/nuclear area percentage calculated with ImmunoRatio program.

**Figure 5:**
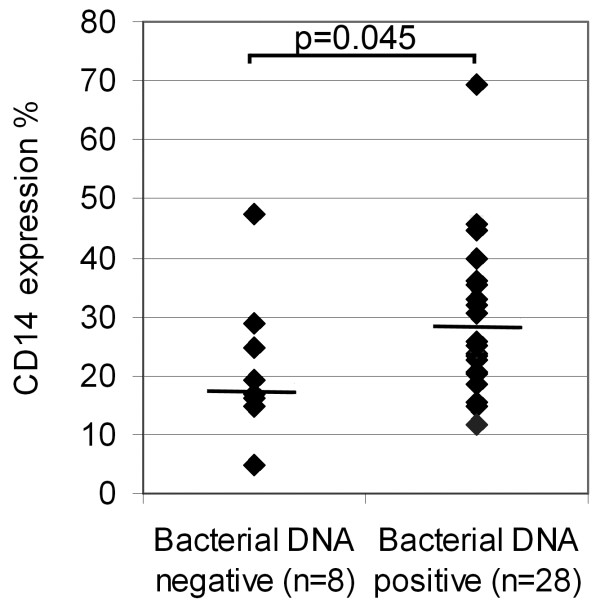
**Immunohistological CD14 expression percentage in bacterial DNA positive and bacterial DNA negative autopsy liver samples calculated with ImmunoRatio software.** Medians are presented as lines.

CD14 percentage differed significantly (p = 0.012) among the controls (median 17.6%), alcoholics (23.3%) and cirrhotics (31.9%). In Post Hoc analyses using LSD correction the CD14 expression percentage was significantly higher in the cirrhotics than in the autopsy controls (p = 0.004) but there were no statistically significant differences between the controls and alcoholics without cirrhosis or between the alcoholics and cirrhotics. Figure 4 presents examples of CD14 staining of control, alcoholic and alcoholic liver cirrhosis cases. Expression of CD14 was higher (p = 0.045) in bacterial DNA-positive liver samples than in DNA- negative samples (Figure 5).

## Discussion

In this study we measured with RT-qPCR the composition of gut microbiota and bacterial translocation into the liver in alcoholic liver cirrhotics and non-cirrhotic alcoholics compared to controls. We found that liver cirrhotics harboured considerably more gram-negative *Bacteroides* and *Enterobactericaea* including *Enterobacter* spp. in their faeces than did the controls. This result is in line with those of other studies [13,14,25] showing increased prevalence of *Enterobactericaea* in the phylum level and also an increase in the counts of *Enterobacter* spp. in the faeces of cirrhotics. Cirrhosis is associated with a decreased conversion of primary to secondary faecal bile acids, which is associated with abundance of major gut microbiome taxa. Bile acids in general, and cholestasis, which is common in cirrhotic patients, may be one mechanistic explanation for the altered microbiota in liver diseases [25]. However, our results do not confirm earlier reports of decreased amounts of gram-negative *Bacteroides* spp. and gram-positive *Bifidobacterium* spp. and increased amounts of gram-positive *Clostridium* spp. in the faeces of cirrhotics [13, 14,].

We also found that the total amount of bacterial DNA in liver was associated with CD14 expression percentage in immunohistochemical stainings. The CD14 expression percentage in cirrhotics was significantly higher due to possible bacterial load compared to that of the autopsy controls. While CD14 macrophages in liver, Kupffer cells, are also capable of recognizing e.g. human cell debris, apoptotic cells and other activating agents [26], they have developed an efficient phagocytic capacity to remove endotoxin from portal circulation. Moreover, since Kupffer cells are continuously exposed to endotoxin and other activators, they show constitutive low-level CD14 activation [27], as is seen in our control liver samples. As far as we know, there are no previous studies reporting the simultaneous measurement of amounts of bacterial DNA and CD14 activity in the liver. Our results suggest that bacteria may have a role as inducers of the CD14 mediated inflammation process that may lead to fibrosis and cirrhosis [28].

It is known that intestinal bacteria contribute to intestinal homeostasis and that alcohol affects microbial populations by disturbing this balance [2]. Alcohol metabolite, acetaldehyde, has a direct effect on gut epithelial cell function by disrupting tight junctions, and may thus enhance bacterial translocation [10] from the intestines. Alcohol ingestion has been reported to correlate with increased levels of bacterial endotoxins [29-31] and peptidoglycan [32] in plasma. Of commensal intestinal bacteria, gram-negative enterobacteria and gram-positive enterococci are the most effective to escape into the organs [11] and are the most commonly found in community-acquired infections in patients with alcoholic liver cirrhosis [33]. Moreover, sepsis is a 20 times more common cause of death in cirrhotics than in general population [34]. Our results showed that *Enterobactericaea* was the most commonly detected in livers of cirrhotics. These differences were not statistically significant, most probably due to small sample size and wide inter-individual variations in bacterial populations. Furthermore, bacterial DNA from *Enterobactericaea* was also detected in ascites.

In cirrhotic patients, attempts have been made to inhibit the growth of gram-negative bacteria in the gut by the use of broad-spectrum antibiotics such as fluorokinones e.g. neomysin, norfloxacin and ciprofloxacin [35,36]. However, long-term use of antibiotics may lead to an increase of pathogenic bacteria in the gut [37] and to increased antibiotic resistance [38,39]. In experimental studies, probiotics *(Bifidobacterium* spp., *Lactobacillus* spp.), have restored normal gut homeostasis and have inhibited excessive growth of gram-negative bacteria [40-42]. In the future, targeted treatments using probiotics or better focused antibiotics against risk pathogens, like *Enterobactericaea*, may be available.

In the present study, relative amounts of commensal gut bacteria and their translocation into liver and ascites was investigated with RT-qPCR. The frequency of bacterial DNA in ascites was 50%, even though none of the volunteers with liver cirrhosis had positive culture or spontaneous bacterial peritonitis. We used universal bacterial primers and probes [22] to amplify all bacterial DNA in the samples. Therefore we believe that our negative cases were genuinely bacterial negative and ascites were sterile in these cases. We did not have control samples from non-cirrhotic ascites, which is a limitation of the study, but except in malignancy, such samples are difficult to obtain. Nevertheless, the same bacteria were found to leak in ascites which were found translocated in liver samples. RT-qPCR provides a fast and accurate tool for the determination of the faecal bacterial composition of clinical patients. Conventional culturing, which is used in many hospital labs, provides only limited opportunities to study certain bacterial strains [16]. Culturing is a relatively slow method and may not provide any information if the bacteria in the samples have been exposed to unfavourable conditions like oxygen or temperature changes leading to their death. Because only 30-40% of the bacteria in the human intestinal tract are culturable, bacterial detection diagnostics can be improved with the DNA-detection based approach [43]. We also found that there were remarkable inter-individual variations in bacterial population ratios, as also reported by earlier studies [44,45].

We have previously shown that e.g. genetic polymorphism in the bacterial CD14 receptor may play a role in susceptibility to cirrhosis [1]. This suggests that susceptibility to alcoholic liver cirrhosis due to bacterial influx into the liver may also be genetically determined. A continuous bacterial translocation from the gut may not be enough per se to cause the development of cirrhosis.

## Conclusions

In conclusion, alcoholic liver cirrhotics had increased amounts of gram-negative enterobacteria in faeces and DNA from enterobacteria was detected in liver and ascites. Total bacterial DNA amount in the liver was associated with immunohistochemical CD14 expression, which was significantly higher in liver cirrhotics than in autopsy controls.

These results suggest that intestinal microbiota and bacterial translocation into the liver may be involved in the pathogenesis of alcoholic liver cirrhosis.

## Abbreviations

RT-qPCR: Real-time quantitative polymerase chain reaction.

## Competing interests

The authors declared that they have no competing interests.

## Authors’ contributions

ST performed the experiments and analyses, wrote the manuscript and helped in the collection of the autopsy samples. TP designed the sample collection and experiments and participated in writing the manuscript. PC participated in the planning of the study and in samples collection from patients. RV and JA provided comments. PK was the initiator of the project and participated in writing the manuscript and collected the autopsy series. All authors have read and approved the final version.

## Pre-publication history

The pre-publication history for this paper can be accessed here:

http://www.biomedcentral.com/1471-230X/14/40/prepub
